# MiR-19a Overexpression in FTC-133 Cell Line Induces a More De-Differentiated and Aggressive Phenotype

**DOI:** 10.3390/ijms19123944

**Published:** 2018-12-07

**Authors:** Giovanna Calabrese, Anna Dolcimascolo, Filippo Torrisi, Agata Zappalà, Rosario Gulino, Rosalba Parenti

**Affiliations:** Department of Biomedical and Biotechnological Sciences, Physiology Section, University of Catania, 95123 Catania, Italy; anna.dolcimascolo@unict.it (A.D.); filippo.torrisi@unict.it (F.T.); azappala@unict.it (A.Z.); rogulino@unict.it (R.G.); parenti@unict.it (R.P.)

**Keywords:** thyroid cancer, follicular thyroid carcinoma, microRNA, miR-19a, aggressiveness, de-differentiation

## Abstract

In recent years, microRNAs (miRNAs) have received increasing attention for their important role in tumor initiation and progression. MiRNAs are a class of endogenous small non-coding RNAs that negatively regulate the expression of several oncogenes or tumor suppressor genes. MiR-19a, a component of the oncogenic miR-17-92 cluster, has been reported to be highly expressed only in anaplastic thyroid cancer, the most undifferentiated, aggressive and lethal form of thyroid neoplasia. In this work, we evaluated the putative contribution of miR-19a in de-differentiation and aggressiveness of thyroid tumors. To this aim, we induced miR-19a expression in the well-differentiated follicular thyroid cancer cell line and evaluated proliferation, apoptosis and gene expression profile of cancer cells. Our results showed that miR-19a overexpression stimulates cell proliferation and alters the expression profile of genes related to thyroid cell differentiation and aggressiveness. These findings not only suggest that miR-19a has a possible involvement in de-differentiation and malignancy, but also that it could represent an important prognostic indicator and a good therapeutic target for the most aggressive thyroid cancer.

## 1. Introduction

Thyroid cancers are considered the most representative malignancies of the endocrine system, affecting about 1% of all newly diagnosed tumor cases with an incidence growing worldwide [1,2]. They comprise some histotypes that can be classified, according to definite histological features, into three different groups: (i) Well-differentiated thyroid carcinomas (WDTCs), (ii) poorly differentiated thyroid carcinomas (PDTCs) and (iii) undifferentiated thyroid carcinomas (UDTCs) [3]. WDTCs include papillary thyroid carcinoma (PTC), which represents about 80% of cases with better prognosis and follicular thyroid carcinoma (FTC) that represents approximately 15% of all thyroid carcinomas, with a less favorable survival rates [4,5,6,7].

PDTCs represents about 0.3% to 6.7% of all thyroid malignancies and display an aggressive clinical behavior, with a poor prognosis [8]. Anaplastic thyroid cancer (ATC) is the most aggressive and lethal form of UDTCs. It shows a quick invasive progression with an early metastatic diffusion, a considerably poorer prognosis and reduced overall survival [9].

MiRNAs, an evolutionally well-conserved class of noncoding RNAs, are capable of controlling gene expression through mRNA cleavage or translational repression [10,11] and their deregulation is involved in the development and progression of several human cancers [12,13,14,15,16]. They work in a complex functional system, in which a precise miRNA regulates hundreds of mRNAs and a single gene is targeted by several miRNAs [17]. MiRNAs regulate various human genes correlated with several cellular processes including proliferation, apoptosis, metastasis, cell immunity and differentiation [18,19,20]. Approximately 2000 miRNAs have been discovered in humans [21] and about 50% have been found in cancer-related genomic regions or fragile sites, confirming their role as drivers of neoplastic alterations [22]. In addition recently, it has been demonstrated that miRNAs expression is correlated with different clinical parameters and their application could be used for cancer diagnosis, prognosis and treatment response [23,24,25].

MiR-19a, one of the seven miRNAs belonging to the miR-17-92 cluster located on chromosome 13q31.3, has been demonstrated to be a tumor-related miRNA involved in cancerogenesis. Several findings have revealed that miR-19a expression is increased in many different types of solid tumors including ATC, as shown in vitro by cell growth induction. Takakura et al., demonstrated that miR-19a is more expressed in thyroid cancer cell lines than in normal primary thyreocytes [26].

The aim of the present study was to evaluate the involvement of miR-19a in promoting de-differentiation towards an aggressive phenotype. For this purpose, we induced miR-19a expression in follicular thyroid cancer cell line (FTC-133) and evaluated its effects on proliferation, cell death and gene expression profile. Our results indicate that miR-19a could contribute to promote a de-differentiation and a more aggressive phenotype of FTC-133 cells. Although further studies are needed, these data could represent a starting point for the development of new therapeutic targets for thyroid carcinomas.

## 2. Results

### 2.1. Quantitation of MiR-19a Level in Basal and Overexpressing Conditions

In order to quantify the expression levels of miR-19a in FTC-133 cells, at both basal and overexpressing conditions, RT-PCR analysis has been performed on total RNA. MiR-19a levels were normalized to those of control U6 snRNA and miRNA levels of cells at 24 and 48 h after overexpression were compared to those of a control group.

**Figure 1:**
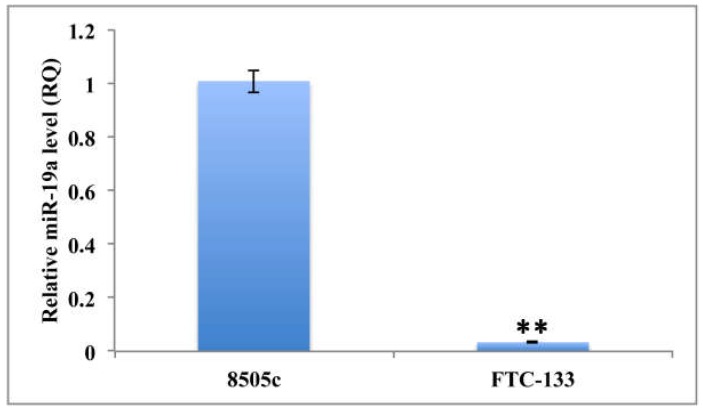
Quantitation of relative miR-19a expression levels on 8505c and FTC-133 cell lines in basal condition. U6 has been used as endogenous control. Student *t*-test *p* value (*p* < 0.0001) is reported and indicates significant difference between the two groups.

**Figure 2:**
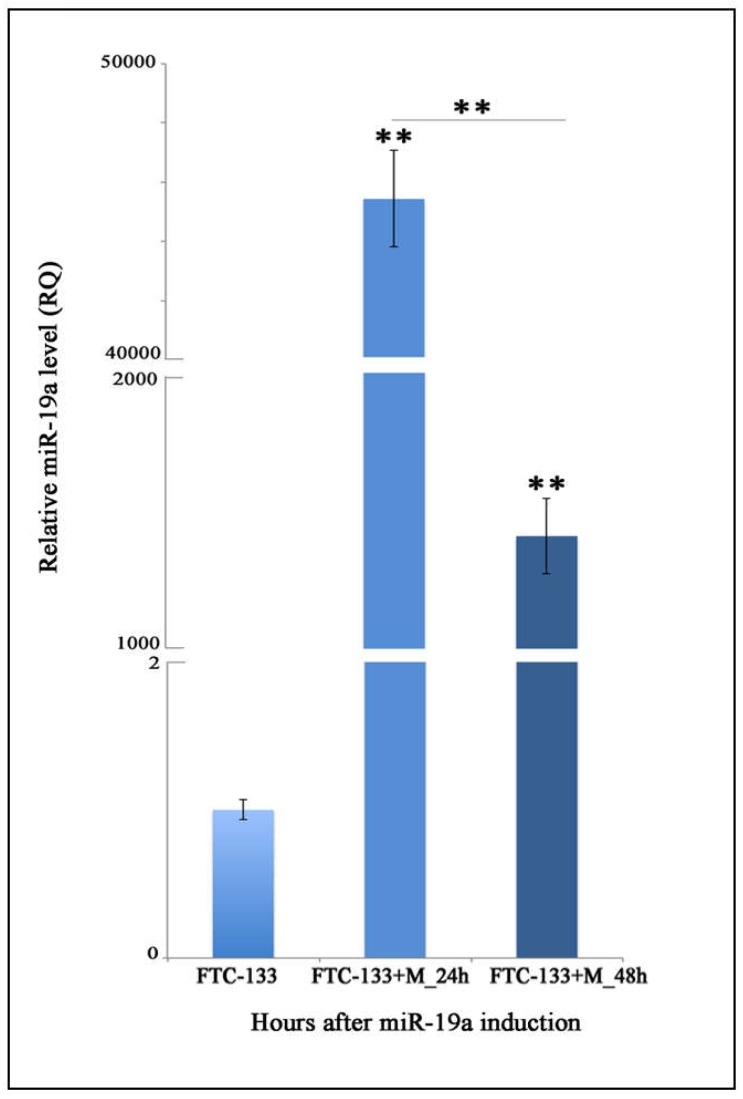
MiR-19a expression levels after miR-mimic overexpression. Relative quantitation (RQ) of miR-19a expression levels time course, using FTC-133 control cells as control group. U6 has been used as endogenous control. ANOVA test *p* value (*p* < 0.0001) is reported and ** (*p* < 0.01) indicates significant differences both between transfected groups and control cultures and transfected groups as reported by the post-hoc test. FTC-133+M: Cells transfected with the miR mimic.

Specifically, to determine miR-19a basal level in the FTC-133 cell line, these cells were compared to anaplastic thyroid carcinoma cells (8505c cell line). Our analysis demonstrated that FTC-133 cells exhibit significantly more reduced expression of miR-19a compared to the 8505c (RQ = 0.0339; ** *p* < 0.01; Figure 1). Furthermore, we quantified miR-19a expression level in FTC-133 cells after miR mimic overexpression and observed a significant increase at both time points (24 and 48 h) compared to the control levels (FTC-133+M_24 h: RQ = 45435.265; FTC-133+M_48 h: RQ = 1389.312; ** *p* < 0.01; Figure 2) even if in FTC-133+M_24 h the miR-19a level was significantly higher (Figure 2; ** *p* < 0.01).

### 2.2. MiR-19a Overexpression on FTC-133 Induces Phenotypic Changes on Cell Morphology

**Figure 3:**
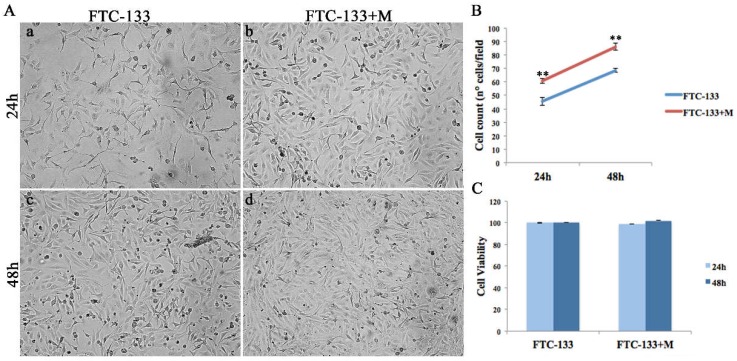
MiR-19a mimic overexpression effects on FTC-133 morphology, proliferation and cell viability. (**A**) Morphological analysis of control (**a**,**c**) and miR-19a mimic overexpressing (**b**,**d**) FTC-133 cells, at 24 and 48 h post-transfection. (**B**) Cell count of control cells (blue) and miR-19a mimic overexpressing cells (red), 24 and 48 h after overexpression. Student *t*-test *p* value (*p* < 0.0001) indicates significant differences between miR-19a mimic transfected groups and control samples. (**C**) MTT assay assessed on FTC-133 and FTC-133+M, 24 and 48 h post-transfection.

The morphological analysis of miR-19a mimic overexpressing FTC-133 cells showed phenotypic changes at both analyzed time points (24 and 48 h) (Figure 3Ab,d) compared to the control (Figure 3Aa,c). Specifically, FTC-133 cells overexpressing the miR-19a mimic showed a less starry and elongated shape and an increased proliferation compared to the control cells (Figure 3Aa–d).

### 2.3. MiR-19a Overexpression on FTC-133 Promotes Proliferation and Cell Viability and Reduces Apoptosis

**Figure 4:**
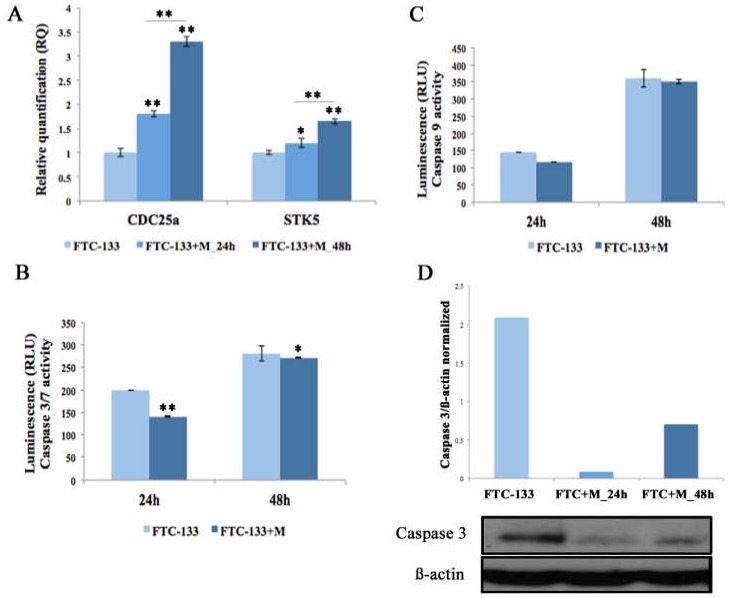
Cell cycle and apoptosis analyses, 24 and 48 h after miR-19a mimic overexpression. (**A**) Relative Quantitation (RQ) of CDC25a and STK5, using FTC-133 cells as control group. GAPDH has been used as endogenous controls. ANOVA test *p* value is reported (*p* < 0.0001) and * (*p* < 0.05), ** (*p* < 0.01) indicates significant differences between groups as reported by the post-hoc test. (**B**,**C**) Caspase-3/7 and Caspase-9 activity is expressed in relative luminescence units (RLU). The x-axis represents FTC-133 and FTC-133+M at 24 h and 48 h post-transfection. Each point indicates the mean and SD of three independent experiments. Student *t*-test value (*p* < 0.001) indicates significant differences between transfected groups and control samples. (**D**) Western blot analysis of Caspase 3 on FTC-133 and FTC-133+M, 24 and 48 h post-transfection. Data show the ratio between intensity of Caspase 3 bands divided by relative ß-actin bands intensity quantified using imageJ software.

To evaluate the effects of miR-19a mimic overexpression on follicular thyroid carcinoma cells we analyzed proliferation, cell viability and apoptosis processes at 24 and 48 h after miR-19a overexpression (Figure 3 and Figure 4).

Both cell proliferation assays, Dapi (data not shown) and Trypan blue dye exclusion staining (Figure 3B), showed that FTC-133 cells overexpressing the miR-19a mimic revealed a significant increase at both time points (24 and 48 h) compared to the control cells (** *p* < 0.01). Cell viability analysis showed that there was only a slight increase between control and miR-19a mimic overexpressing cells at the time point 48 h (Figure 3C). Further, to detect the effect of miR-19a overexpression on cell growth, we analyzed two cell-cycle genes, *CDC25a* and Aurora kinase B (*STK5*) on both FTC-133 cells miR- mimic overexpressing and control cells. *CDC25a* on FTC-133 cells overexpressing miR-19a mimic displayed an evident up-regulation at both time points compared to the control, more marked at 48 h (Figure 4A, ** *p* < 0.01). Similarly, *STK5* showed an increased expression at both times, 24 and 48 h, statistically more significantly at 48h (Figure 4A, 24 h ** *p* < 0.05 and 48 h ** *p* < 0.01).

On the other hand, we performed Caspase 3/7 and Caspase 9 analyses to assess the influence of miR-19a on apoptotic processes at 24 and 48 h after miR mimic overexpression. We showed that the overexpression of miR-19a mimic on FTC-133 cells, at both assessed time points, decreased Caspase 3/7 and Caspase 9 levels (Figure 4B,C) even if the differences were significant only for Caspase 3/7 (Figure 4B, 24 h ** *p* < 0.01 and 48 h * *p* < 0.05). Furthermore, we quantified the endogenous Caspase 3 level by Western blot and confirmed that miR-19a overexpression in FTC-133 reduced protein levels at both time points, especially at 24 h (Figure 4D).

In addition, to confirm the possible role of miR-19a overexpression on proliferation and cell survival, we performed miR-19a inhibition on 8505c cells and evaluated its effects at 24 and 48 h after down-regulation. Cell proliferation assay performed 24 and 48 h after miR-19a inhibition on 8505c cells showed a marked decrease only at 48 h (Supplementary Figure S1A, ** *p* < 0.01). MTT assay assessed on miR-19a down-expressing 8505c cells showed a statistically significant decrease of cell viability at both analyzed time points (Supplementary Figure S1B, 24 h * *p* < 0.05, 48 h ** *p* < 0.01). Caspase 3/7 and Caspase 9 analyses performed 24 and 48 h after miR-19a inhibition displayed a significant increase of both caspases only at 48 h (Supplementary Figure S1C,D Caspase 3/7 48 h ** *p* < 0.01, Caspase 9 48 h * *p* < 0.05).

### 2.4. MiR-19a Overexpression on FTC-133 Alters the Expression of Genes Related to Thyroid Differentiation and Poor Prognosis

To assess if miR-19a up-regulation in FTC-133 altered the expression profile of genes correlated with thyroid cells differentiation and poor prognosis, such as in 8505c cells, we performed qRT-PCR with thyrocyte-specific markers, including thyroid stimulating hormone receptor (*TSHr*), thyroglobulin (*Tg*), transcription termination factor 1 (*TTF1*), paired box gene 8 (*Pax8*), and poor prognosis genes, including Cadherin-1 (*CDH1*) and S100 calcium-binding protein A4 (*S100A4*).

**Figure 5:**
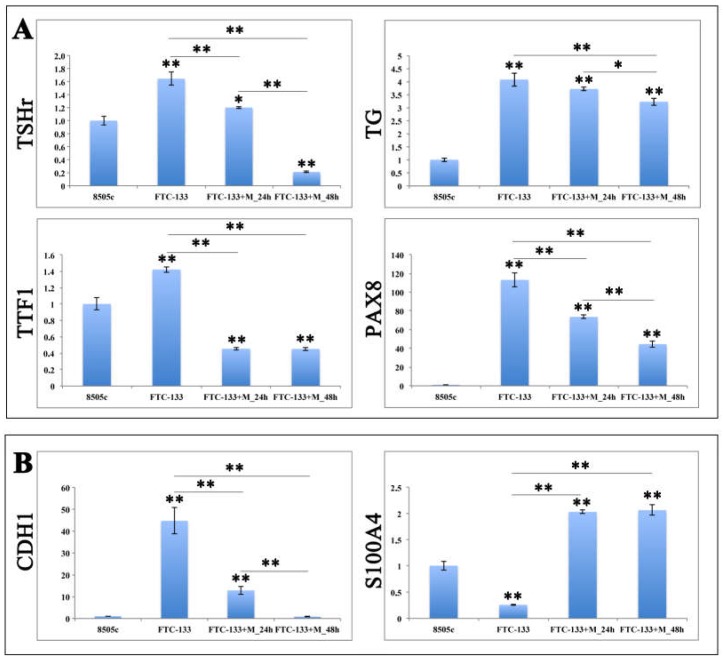
Gene expression analyses with (**A**) thyroid cells differentiation and (**B**) poor prognosis related genes on miR-19a mimic overexpressing FTC-133 cells. Relative Quantitation (RQ) of genes involved in thyroid cells differentiation and poor prognosis using 8505c cells as control group. qRT-PCR has been performed for the mRNA of *Tg*, *TSHr*, *Pax8*, *TTF1*, *CDH1* and *S100A4*. Glyceraldehyde-3-Phosphate Dehydrogenase (GAPDH) was used as endogenous controls. ANOVA test *p* value is reported (*p* < 0.0001) and * (*p* < 0.05), ** (*p* < 0.01) indicates significant differences between groups as reported by the post-hoc test.

The gene expression profile of FTC-133 cells, overexpressing miR-19a mimic, displayed a statistically significant reduction of genes related to thyroid cells differentiation at 24 and 48 h after miR mimic overexpression (Figure 5A). Specifically, *TSHr*, *Tg* and *Pax8* expression levels were significantly more down regulated at 24 h than at 48 h when compared to the control, while the levels of *TTF1* were equally reduced at both time points. Moreover, FTC-133 cells overexpressing the miR-19a mimic exhibited an higher expression levels of *Pax8* and *Tg* at both 24 and 48 h, compared to the 8505c group, although these were markedly down regulated compared to the FTC-133 control. On the other hand, *TSHr* and *TTF1* were significantly lower than in 8505c, except for *TSHr* at 24 h (Figure 5A).

The expression profile of *CDH1* and *S100A4*, two poor prognosis genes involved in cell migration, proliferation and invasion, exhibited a striking decrease for *CDH1* in FTC-133 transfected cells overtime, compared to the FTC-133 control (Figure 5B; ** *p* < 0.01) and a comparable expression with 8505c cells at 48 h; while *S100A4* revealed an evident constant increased at 24 and 48 h, statistically significant compared to both FTC-133 control and 8505c cells (Figure 5B; ** *p* < 0.01).

## 3. Discussion

In the last years, it has been shown that miRNAs deregulation is involved in development and progression of several types of cancers including B cell chronic lymphocytic leukemia [27], breast tumor [28], lung cancer [29], gastric tumor [30], colorectal cancer [31], glioblastoma [32], prostate tumor [33] and thyroid carcinoma [34,35]. MiR-19a is the main component responsible for the oncogenic activity of miR-17-92, promoting the growth and migration of tumor cells [36].

MiR-19a is overexpressed in a great quantity of solid tumors, such as bladder cancer [37], myeloid leukemia [38], colorectal cancer [39,40], gastric cancer [41], multiple myeloma [42], lung cancer [43], ovarian cancer [44], glioma [45,46], clear cell renal cell carcinoma [47], and anaplastic thyroid carcinoma [23]. Moreover, the miR-19a oncogenic role was described in laryngeal squamous cell carcinoma, correlating with lymph node metastasis, poor differentiation and diminished overall survival [48]; it also promotes cell proliferation of bladder cancer cells [37] and it is involved in growth, invasion and migration of non-small cell lung cancer [49].

MiR-19a is also involved in tumor development by promoting cell proliferation and migration, as well as by reducing apoptosis. For example, Liu et al. reported that miR-19a promotes cell proliferation and migration in colorectal cancer cells and accelerates tumor growth in xenograft mice [39]; Fu et al., demonstrated that miR-19a overexpression exerts its oncogenic activity by stimulating cell proliferation, cycle, growth and migration [50]. Finally, Paiva et al., suggested that the development of thyroid cancer derived from miR18a and miR19a altered expression depends primarily on cell-specific proteins [51].

Although miR-19a has also been found to be altered in ATC, its functional involvement is still poorly investigated.

ATC, which is the most aggressive and fatal thyroid neoplasia characterized by uncontrolled growth, high invasiveness and not responsive to surgery and radioiodine, originates from the de-differentiation of a pre-existing well-differentiated or poorly differentiated thyroid carcinoma [52,53,54]. Among conditions and factors promoting ATC, several genetic alterations have been described including mutations in tumor suppressor *TP53*, MAPK signaling (*RAS* and *BRAF* genes), PI3K signaling (*PIKCA* and *PTEN*), and Wnt signaling (β-catenin and *APC*) [55,56,57,58,59].

The BRAF1799T>A mutation, causing the BRAFV600E oncoprotein, is very frequent in well differentiated thyroid cancer and in ATC derived from PTC [60]. Fuziwara et al., in their study demonstrated that BRAFV600E activates miR-17-92 via upregulation of Notch signaling [61].

However, given the severity of this neoplasm, because the scenario that characterizes ATC is still largely unknown, it becomes essential to evaluate the involved functional aspects to identify new and more promising therapeutic targets.

In this study, we firstly investigated miR-19a overexpression in FTC. Thus, to assess the association of miR-19a overexpression with de-differentiation and malignancy of thyroid cancer, we induced miR-19a expression in a well-differentiated thyroid tumor cell line, FTC, and analyzed the expression profile of specific markers of clinical relevance in the poor prognosis and thyroid cells differentiation. We found that miR-19a overexpression significantly increases the cell proliferation and reduces apoptosis consistently with the miR-19a role in other cancers [43,62]. Since it is known that *PTEN* [63] is one of the predicted targets of miR-19a, it is also possible that miR-19a overexpression might be associated with *PTEN* inactivation inducing cell proliferation in FTC-133, while its inhibition promotes *PTEN* activation inducing cell growth reduction. In a recent study, Frisk et al. have suggested that *PTEN* suppression is associated in extremely malignant or late-stage thyroid tumors, including ATC [64]. These results suggest that miR-19a leads to the poor prognosis in follicular thyroid cancer cells through stimulating cell proliferation.

Furthermore, we demonstrated that miR-19a overexpression correlates with the loss of the most differentiated phenotype, as suggested by the marked down regulation of *TSHr*, *Tg*, *TTF1* and *Pax8*, specific genes and transcription factors associated to the thyroid cells differentiation [65,66,67,68]. *TTF1* (also known as *Nkx2-1*) and *Pax8* are two key genes for thyroid gland development and morphogenesis [69] and their expression is critical for the thyroid differentiated phenotype and for the transcriptional activation of the differentiation markers, such as *Tg* and *TSHr* [67,70,71]. Several studies reported that the neoplastic thyroid cells de-differentiation is correlated with the reduction or even the loss of specific thyroid genes, *Tg* and *TSHr*, and their transcription factors, *TTF1* and *Pax8* [72,73]. Our data support the idea that the decrease or loss of expression of thyroid cells differentiation markers is associated with the most de-differentiated phenotype of thyroid neoplasia.

Finally, we demonstrated that miR-19a is involved in malignant phenotype and aggressiveness as shown by the consistent increase *S100A4*, a main component of S100 family proteins, strictly associated to the malignant phenotype and metastatic behavior of various tumors [74,75,76] and decrease of *CDH1*, an E-cadherin, which down expression is found in several types of cancer and relates with infiltrative and metastatic behavior [77,78,79,80].

In conclusion, the present work supports the idea that the miR-19a may be involved in de- differentiation and in the “poorer prognosis” of thyroid cancer, thus suggesting that this miRNA could represent a good prognostic indicator and a valid therapeutic target for highly malignant anaplastic tumors, although additional studies on human samples are also needed in order to prove the association of miR-19a expression with the relevant clinicopathological factors and prognosis.

## 4. Material and Methods

### 4.1. Cell Culture

FTC-133 (Sigma-Aldrich, Italy) and 8505c (Sigma-Aldrich, Italy) human cell lines were used in this analysis. FTC-133 cells were cultured in DMEM: Ham’s F12 (1:1) (Sigma-Aldrich, Italy) supplemented with L-Glutamine 2 mM (Euroclone, Italy), penicillin/streptomycin/amphotericin (PSA) (Euroclone, Italy), and 10% Fetal Bovine Serum (FBS) (Sigma-Aldrich, Italy), while 8505c cells were grown in EMEM (Sigma-Aldrich, Italy) containing 1% of non-essential amino acids, L-Glutamine 2 mM, PSA and 10% FBS. Cells were maintained in a humidified environment at 37 °C and 5% CO_2_. The medium was replaced twice a week and cells were split at about 80% to 90% of confluence.

### 4.2. MiRNA Mimic/Inhibitor Transfection on FTC-133 and 8505c Cells

FTC-133 and 8505c cells were transfected, respectively, with specific mirVana miRNA mimic and inhibitor (hsa-miR-19a-p3; Life Technologies, Camarillo, CA, USA) in accordance with the manufacturer’s instructions. Briefly, both Lipofectamine RNAiMAX reagent (ThermoFischer Scientific, Italy) and miRNA (10 µM initial concentration) were dilute in Opti-MEM Medium (ThermoFischer Scientific, Italy). MiRNA solution was supplemented with an equivalent volume of Lipofectamine RNAiMAX reagent (1:1 ratio) and the resulting mix was incubated for 5 min at room temperature. After, the mix (miRNA mimic /inhibitor final concentration 0.1 µM) was added to 0.25 × 10^6^ cells/6-well, as reported by the manufactures protocol. Lastly, transfected cells were incubated in a humidified environment at 37 °C and 5% CO_2_/95% air atmosphere for 24 and 48 h. Cells transfected only with the transfecting agent were used as control. Cell morphology was analyzed, and digital images were acquired using a Leica DMI4000B microscope.

### 4.3. Cell Proliferation Analysis Through DAPI and Trypan Blue Staining

For DAPI staining, FTC-133 cells were fixed in 4% PFA for 15 min and permeabilized in 0.3% Triton X-100 for 5 min. Successively, the cells were washed three times with PBS and the nuclei stained with DAPI (1:5000) in PBS for 5 min. Slides were mounted in fluorescent mounting medium Permafluor (Thermo Scientific) and digital images were acquired using a Leica DMI4000B fluorescence microscope. At least five images from each sample were taken for the count. Cell count analysis has been performed using Fiji image recognition software. For trypan blue dye exclusion staining we added 0.1 mL of trypan blue solution (0.4% in PBS) to 0.1 mL of cells, and after loaded on a hemacytometer, we counted immediately under a microscope at low magnification the number of blue staining cells and the number of total cells.

### 4.4. Measurement of Cell Viability Through MTT Assay

The cell viability of the two different cell samples, treated with only transfecting agent and treated with miR mimic or miR-inhibitor, was measured using an MTT [3-(4,5-dimethylthiazol-2-yl)-2,5-diphenyltetrazolium bromide] (Sigma-Aldrich, Italy) assay. Specifically, 24 and 48 h after cell transfection the medium from each well was removed and replaced with 200 µL of MTT solution (1 mg/mL in FBS-free medium). Following 2 h incubation at 37 °C and 5% CO_2_ MTT solution was removed, each well was washed two times using cold PBS 0.01 M, and the formed crystals were melted using 200 μL of DMSO. Next, the absorbance at 570 nm was read using a synergy HT plate reader (BioTek Instruments, Inc., VT, United States).

### 4.5. Caspase-3/-7 and Caspase-9 Analyses

Briefly, the Caspase-Glo^®^ 3/7 or 9 Buffer and lyophilized Caspase-Glo^®^ 3/7 or 9 Substrate (Promega, Milan, Italy) as well as the 96-well plates containing cells were equilibrated at room temperature (10 min). Caspase-Glo^®^ 3/7 or 9 Buffer was mixed with Caspase-Glo^®^ 3/7 or 9 Substrate by inverting the contents. A total of 50 µL of the mixed solution was added to an identical volume of each sample, in agitation by using a plate shaker at 500 rpm for 1 min, and the plates left to incubate at room temperature for 1 h. A blank reaction, consisting of Caspase-Glo^®^ 3/7 or 9 Reagent and only vehicle was used as control. The luminescence of each sample was read using a synergy HT plate reader (BioTek Instruments, Inc., VT, United States).

For Western blot analysis, cell pellets were homogenized in lysis buffer (Tris-HCl pH 7.4, 1% Triton X100, NaCl 150 mmol/L and EDTA 1 mmol/L) supplemented with a cocktail of protease inhibitors (1:100, Sigma). For the quantification, 40 µg of protein were separated on a precast 4% to 20% tris-glycine gel (Thermo Scientific, Rockford, IL, USA) and transferred to a nitrocellulose membrane. After blocking the following primary antibodies: Mouse caspase 3 (Santa Cruz, 1:500) and mouse ß-actin (Santa Cruz, 1:500). After three washes in TBST, the membranes were incubated with anti-mouse HRP-conjugated secondary antibody (Thermo Scientific group; 1:6000) for 1 h at RT. Peroxidase activity was developed by enhanced chemiluminescent substrate (Pierce Biotechnology Inc., Thermo Scientific) and visualized by autoradiography. The density of each band was quantified using ImageJ analysis software and to ß-actin levels measured in the same membrane.

### 4.6. qRT-PCR

For qRT-PCR analyses total RNA from FTC-133 cells was isolated 24 and 48 hours after miR mimic transfection using RNeasy Mini Kit (Qiagen, Germantown, MD, USA) and quantified as previously described [81]. Three independently cultured samples were used for each of the two time points. cDNA was synthesized from 1 µg of total RNA using ImProm-II Reverse Transcription System (Promega, Milan, Italy). qRT-PCR was performed using SYBR Green method on a 7900HT Real Time PCR using manufactures protocol (Applied Biosystems). Specific primers for each of the investigated molecular endpoint were designed using primer blast and selecting exon-exon junctions on mRNA as target region for annealing. Each sample was tested in triplicate and gene expression was assessed using the 2^−ΔΔCt^ method. RNA from control cells was used as reference for relative expression quantitation. The following primers for qRT-PCR were used: *CDH1* F: TGCCCAGAAAATGAAAAAGG, R: GTGTATGTGGCAATGCGTTC; *S100A4* F: GTGACGCCCTGTCTCTAAGC, R: ATAGCAACAGCGTGTGCAAG; *Tg* F: AACCCCATTGTGTTCTCAGC, R: CATTAGCCCAGGCTTCAGAG; *Pax8* F: AAGGTGGTGGAGAAGATTGG, R: GCTGCTCTGTGAGTCAATGC; *TSHr* F: ATGGGGATGTACCTGCTCCT, R: AATGAGATTGGGGCCATGCA; *TTF1* F: GCCTCAGTCAGAATCCCACC, R: GGTATGCACTCTCGAGGCTC; *CDC25a* F: GGCAAGCGTGTCATTGTTGT; R: GGCTCACAGTAAGACTGGCA; *STK5* F: GTGTACTTGGCTCGGGAGAA; R: CAGCTCTTCTGCAGCTCCTT; *GAPDH* F: GGAAGGTGAAGGTCGGAGT, R: TGGGTGGAATCATATTGGAA. 

Glyceraldehyde 3-Phosphate Dehydrogenase (GAPDH) was used as a reference gene. Expression Suite Software v1.1 was used for data analysis.

The expression levels of miR-19a in FTC-133 after overexpression were calculated as described by Yang and colleagues [82,83]. The following primer sequences were used: miR-19a, RT: 5’-GTCGTATCCAGTGCAGGGTCCGAGGTATTC GCACTGGATACGACTCAGTTT-3’; F: 5’-CTGGAGTGTGCAAATCTATGC-3’; R: 5’-GTGCAGGGTCCGAGGT-3’; *U6*, RT: 5’-AAAATATGGAACGCTTCACGAATTTG-3’; F: 5’-CTCGCTTCGGCAGCACATATACT-3’; R: 5’-ACGCTTCACGAATTTGCGTGTC-3’. The relative expression level of miR-19a was calculated using 2^−ΔΔCt^ method, with the CT values normalized using U6 as an internal control.

### 4.7. Statistical Analysis

Statistical analysis was performed by Student *t*-test and one-way ANOVA. Tukey HSD (Honestly Significant Difference) method was used as a post hoc test when the ANOVA reported statistically significant differences, to evaluate the differences between the individual time-points or treatment groups.

## Supplementary Materials

The following are available online at http://www.mdpi.com/1422-0067/19/12/3944/s1, Figure S1: MiR-19a inhibitor effects on 8505c proliferation, cell viability and apoptosis.
