# Supplementary material for: MiR-19a Overexpression in FTC-133 Cell Line Induces a More De-Differentiated and Aggressive Phenotype
Source: Int J Mol Sci. 2018 Dec 7;19(12):3944. doi: 10.3390/ijms19123944 (PMC6320980; doi:10.3390/ijms19123944)
Supplement: Supplementary file 1 [file ijms-19-03944-s001.pdf]

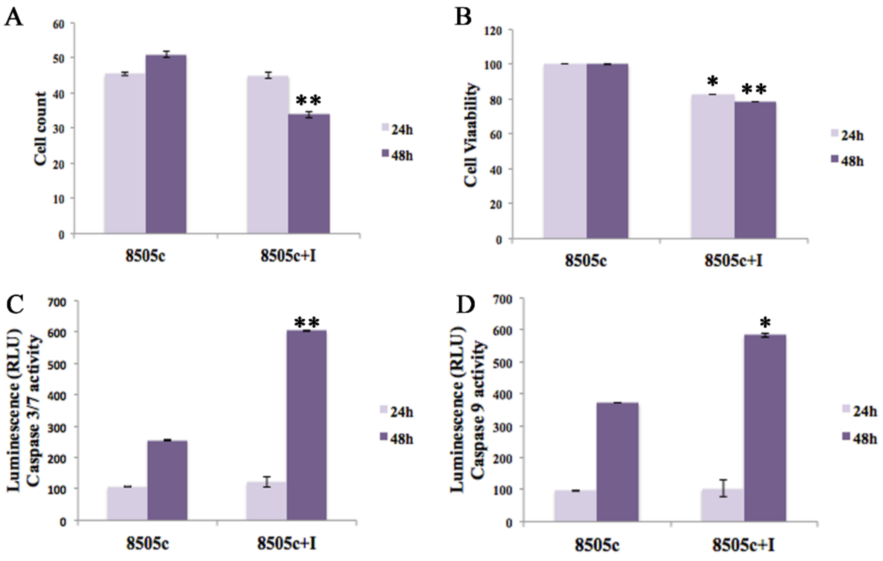

Supplementary Figure 1. miR-19a inhibitor effects on 8505c proliferation, cell viability and apoptosis. (A) Cell count of 8505c and 8505c+I cells, at 24 and 48 hours after inhibition. Student t-test p value ($p<0.0001$) indicates significant differences between miR-19a inhibitor transfected groups and control samples. ANOVA test p-value is showed, ** ($p<0.01$) indicate significant differences between transfected groups at 24 and 48h and control sample as reported by the post-hoc test. (B) MTT assay assessed on 8505c and 8505c+I cells, 24 and 48h post-transfection. (C, D) Caspase-3/7 and Caspase-9 activity assessed on control and transfected cells, after 24 and 48h. Activity is expressed in relative luminescence units (RLU). The x-axis represents time in hours post-transfection. Each time point indicates the mean and SD of three independent experiments. ANOVA test p-value is showed, * ($p<0.05$) and ** ($p<0.01$) indicate significant differences between transfected groups at 24 and 48h and control samples as reported by the post-hoc test.
